# Extended reality in language learning: A cognitive affective model of immersive learning perspective

**DOI:** 10.3389/fpsyg.2023.1109025

**Published:** 2023-02-03

**Authors:** Yuying Zhi, Lihuan Wu

**Affiliations:** ^1^Department of English, College of Foreign Languages, University of Shanghai for Science and Technology, Shanghai, China; ^2^School of Foreign Languages, East China University of Science and Technology, Shanghai, China

**Keywords:** extended reality (XR), language learning, Cognitive Affective Model of Immersive Learning (CAMIL), virtual context, cognitive factors

## Abstract

A surge in the interest and implementation of extended reality (XR)-based lessons in language learning has resulted in many related studies. Recent reviews that summarized these studies and the previous studies focus on the technologies used in language-learning settings or the different ways of incorporating XR tools in language-learning activities. However, less work has been done to synthesize XR-based language-learning studies from a language-learning theory perspective. Thus, this study delineates the contour of scholarly literature on XR in language learning using the Cognitive Affective Model of Immersive Learning (CAMIL). The model contains six affective and cognitive factors that lead to XR-based learning: interest, motivation, self-efficacy, embodiment, cognitive load, and self-regulation. This model was adopted in the current study to systematically synthesize the findings from primary studies published between 2017 and 2022 to construct XR explanations on language learning from a cognitive theory perspective. Studies published in 12 indexed privileged journals in the language education and technology field on XR in language learning were reviewed. The results showed that the factors in the CAMIL led to factual, conceptual, and procedural knowledge acquisition and transfer. This study provides some insights into understanding the cognitive outcomes of XR-based language learning by analyzing the findings from previous studies. Suggestions for future studies are proposed in this study.

## 1. Introduction

Recently, technology applications have broadened to support language learning. One theme that has attracted the most attention is the use of extended reality (XR), which is an umbrella term for virtual reality (VR), augmented reality (AR), and mixed reality (MR) technologies, in language learning. Studies showed that XR has enriched the teaching and learning processes (Cai et al., 2021). In addition, XR has been reported to provide learners with a real-like physical world that could activate learners’ cognitive language-learning system to increase learning interest and motivation or to foster learners’ self-efficacy and self-regulation (Dolgunsöz et al., 2018; Xie et al., 2019; Ebrahimi, 2022). Recent reviews summarized previous studies on implementing XR in language learning; however, less work has been done on the existing studies from a language-learning theory perspective.

In this study, previous studies on XR use in language learning are reviewed from the perspective of the Cognitive Affective Model of Immersive Learning (CAMIL). Accordingly, XR use in language learning is summarized and CAMIL is illustrated. The previous studies are analyzed based on their technological factors, XR affordance, cognitive factors, and learning outcomes. The analysis indicates that the current research on XR use in language learning tends to focus on isolated factors and that a systematic illustration of the relationship among different factors is lacking. Therefore, this study bridges the gap in XR use in language-learning research by adopting a CAMIL perspective. Finally, future directions primarily related to XR application in language learning against language-learning theories are discussed.

## 2. Extended reality in language learning

The term “XR” is an umbrella term that covers various simulation-based technologies such as AR, VR, and MR. The nature of XR permits users to interact with virtual and real objects in the same space, thus creating a new learning experience (Chen and Chan, 2019).

During the last decades, XR technologies have been applied to several educational fields, such as science and mathematics in schools and in tertiary education. As a non-technical subject, language learning can also benefit from XR technologies. For example, XR-assisted language learning could activate learners’ motivation and collaboration (Bacca Acosta et al., 2014). Similarly, existing studies showed that XR could trigger learners’ cognitive ability and improve their concentration skills (Saidin et al., 2015). Technological progress creates an appropriate immersion system that provides the learners’ real-like learning context and is a core component of language learning.

The existing reviews on XR-based language learning range from how XR technology was used to obtain the learning outcome benefits from XR technology. Parmaxi’s (2020) review focused on how VR is adopted in language learning by categorizing typical features employed in VR-based language-learning activities. Huang et al. (2021) also systematically reviewed AR- and VR-involved language-learning studies, and they concluded that XR technology could enhance learners’ motivation and improve learning outcomes. Especially, Cai et al. (2021) meta-analyzed AR-based language-learning studies to establish the relationship between learning outcomes and motivation. To synthetically analyze the VR-enhanced language-learning studies, Punar Özçelik et al. (2022) reviewed existing studies from three aspects, namely, the research features, the research results, and the theories behind AR. In their review, constructivism sociocultural theory and connectivism are proposed as the guiding theories while applying AR in language learning.

In summary, previous reviews synthesized XR-based language-learning studies from general claims to specific effects of one factor and from the features of XR-based learning activities to the learning effects in a different dimension. However, how to systematically illustrate an XR-involved language-learning process with learning theories is still insufficiently understood.

## 3. Cognitive affective model of immersive learning

The CAMIL was proposed by Makransky and Petersen (2021) to provide a theoretical framework for understanding XR-involved language learning. CAMIL was established with four aspects: technological factors, XR affordance, cognitive factors, and learning outcomes (Makransky and Petersen, 2021). The general theoretical framework of CAMIL suggests that the language-learning methods interact with media, which is also the learning context.

The CAMIL model is composed of technological factors, XR affordances, cognitive factors, and learning outcomes. According to Makransky and Petersen (2021), the technological factors in CAMIL include immersion, representational fidelity, and control factors. XR affordance in learning with XR is the presence and agency and the six cognitive factors in CAMIL are interest, motivation, self-efficacy, embodiment, cognitive load, and self-regulation. The learning outcomes are categorized as factual knowledge, conceptual knowledge, procedural knowledge, and transfer of learning. How the affordance factors trigger the cognitive factors is the core of CAMIL.

Extended reality affordance explains how technologies are applied in language learning in the virtual context. In CAMIL, the sense of presence and agency is highly psychological. Presence refers to the learners’ feelings of being there. Both physical and social presence can be achieved using CAMIL. While physical presence refers to the situation that the virtual physical objects are experienced as actual physical objects, the virtual social actors are experienced as actual social actors in the virtual context. Both physical and social presence are the psychological states that enhance the learning context and interaction or collaboration in the learning process (Lee, 2004). Agency in CAMIL refers to the feeling of controlling and generating actions in a virtual context. The learners can control their actions in a virtual context and exert that control over environmental parameters (Johnson-Glenberg, 2019). Thus, XR affordance in CAMIL can reflect both the learners’ presence and self-control in the virtual learning context.

The six cognitive factors are the important components of CAMIL. Interest is a psychological construct representing a relationship between a learner and a specific learning content. Focused attention and cognitive reaction are activated by entering the virtual context (Hidi and Renninger, 2006). Motivation is the learners’ engagement in an activity for the associated built-in satisfaction (Deci and Ryan, 2015). Makransky and Lilleholt (2018) proposed that the higher the presence in XR-based learning, the higher the motivation. Self-efficacy is the learners’ perceived capabilities for learning. The learners in a virtual context have a high sense of presence and agency, which lead to the feeling that they control their activities. Embodiment refers to the sensation that the learners are inside the virtual context and their experience of having and controlling a virtual body (Kilteni et al., 2012). This virtual body can be influenced by the ability to control the actions and the possibility of feeling the sensorial events directed to the body (Makransky and Petersen, 2021). In the virtual world, cognitive load describes how the learners process information during learning, and self-regulation refers to the learners’ ability to manage their behavior in the virtual world to maintain learning focus and undertake learning tasks (Makransky and Petersen, 2021).

## 4. Methods

Extended reality technology used in language learning is a recent topic and it would be beneficial to know if the current literature can enhance the use of XR technology in language learning. Therefore, this study provides a mini-review and analyzes the existing research on XR in language learning from a CAMIL perspective.

Previous studies that reviewed the current research were collected from academic journals with high-impact factors published from 2017 to 2022. Unlike preceding reviews that the author(s) collected from the existing studies by searching the database, the current study analyzed previous XR-related reviews (Parmaxi, 2020; Huang et al., 2021; Makransky and Petersen, 2021; Punar Özçelik et al., 2022) and meta-analysis studies (Cai et al., 2021) to identify primary academic journals. In addition, the reference lists in these reviews were analyzed to avoid omitting any relevant studies. Finally, 12 academic journals were selected as the database to collect relevant articles: (1) *Journal of Computer Assisted Learning*; (2) *British Journal of Educational Technology*; (3) *ReCALL*; (4) *Frontiers in Psychology*; (5) *Language Learning and Technology*; (6) *Computer Assisted Language Learning*; (7) *Computers and Education*; (8) *Bilingualism: Language and Cognition*; (9) *Journal of Language and Linguistic Studies*; (10) *VR*; (11) *Cognition*; and (12) *Interactive Learning Environments*. All the articles published in the above journals from 2017 to 2022 were manually examined. The initial examination yielded 38 XR-related study articles, and among them, only 10 articles empirically studied XR-involved language learning. Therefore, the current study only reviewed the 10 empirical studies.

## 5. Results and discussion

**FIGURE 1:**
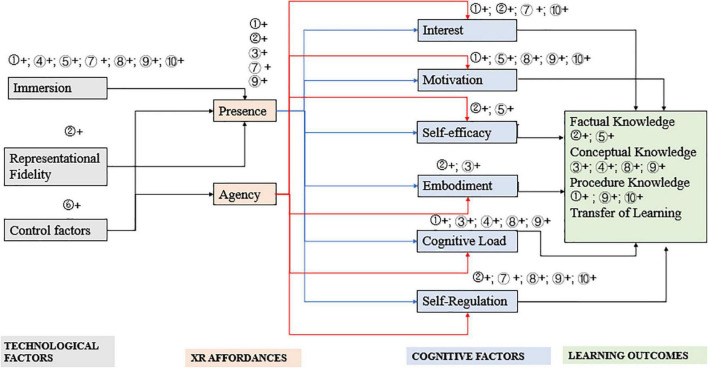
Features of the 10 reviewed studies within Cognitive Affective Model of Immersive Learning (CAMIL). The serial number in this figure refers to the 10 studies stated above, and “+” refers to positive influence.

As a result of the examination, 10 studies were reviewed using the CAMIL model: (1) Dolgunsöz et al. (2018), (2) Xie et al. (2019), (3) Jain et al. (2020), (4) Chen et al. (2021), (5) Fuhrman et al. (2021), (6) Nicolaidou et al. (2021), (7) Wan Daud et al. (2021), (8) Ebrahimi (2022), (9) Lin et al. (2022), and (10) Tai (2022). The features of the above studies are shown in Figure 1.

As shown in Figure 1, studies on XR used in language learning range from technological factors, XR affordance, cognitive factors, and learning outcomes.

### 5.1. Findings related to technological factors

Concerning the technological factors in XR-related language learning studies, seven studies analyzed the immersive media of XR (Dolgunsöz et al., 2018; Jain et al., 2020; Chen et al., 2021; Nicolaidou et al., 2021; Ebrahimi, 2022; Lin et al., 2022; Tai, 2022). The studies showed that the realistic environment created by XR technologies could trigger the learners’ cognitive factors, such as interest or motivation in language learning.

The learners like being in the task environment from the first-person view. The learners’ feeling of involvement and their use of immersive XR applications can effectively support their writing performance, listening comprehension, and vocabulary acquisition. The immersive feature of XR technology matched the language-learning theories in that language is learned from the usage in the context. According to the constructivist learning theory, learning by engaging learners in a learning environment where they can connect their classroom learnings with the real world is one of the essential components of successful language learning.

Compared to immersion in XR use in language learning, representational fidelity, and control factors were less investigated, with each feature mentioned only by one study. However, representational fidelity and control factors in XR technology are also important for driving the physical presence in the virtual environment (Makransky and Petersen, 2021).

### 5.2. Findings related to XR affordance

Of the 10 XR affordance review studies, 9 focused on presence. According to Makransky and Petersen (2021), the immersion of XR technology deals with the extent of the sensory information presented. Virtual physical objects are similar to actual physical objects in sensory ways, which is also the psychological state of the learners in XR language learning. Thus, the rich and integrated sensory provided by the XR technology may promote learners’ language learning, such as vocabulary acquisition and the fully immersive context facilitates learners’ comprehension of listening materials. The amount of control over technical factors like the environmental sensors and the degree to which the learners could modify the objects in the visual world could also influence the feeling of being there. Moreover, representational fidelity also affects presence by displaying a realistic environment and smoothness of view change. However, these two points were less examined in the reviewed studies.

Agency in XR affordance in language learning is not studied in the 10 reviewed articles. Agency in a virtual context is the learners’ control over the environmental parameters, which is a more technical issue during language learning (Johnson-Glenberg, 2019). It is one of the possible reasons why the agency is less investigated in XR-based language learning.

### 5.3. Findings related to cognitive factors and learning outcomes

Ten reviewed studies examined all six cognitive factors in CAMIL. Interest, motivation, cognitive load, and self-regulation were thoroughly discussed in the previous XR-based language-learning studies. Dolgunsöz et al. (2018) stated that most learners have never experienced XR technology but have heard about it, which piqued their interest. Xie et al. (2019) found that the unknown information offered by XR facilitated learners to discover more novel information about the content, which drove the learners to explore additional information on the topic. The learners not only learned the language itself but the culture, and the latter inevitably brought out newer content, which is the embodiment factor of CAMIL (Xie et al., 2019). Both focused attention and affective reaction are activated in the virtual context that matches the psychological construct, which is the relationship between the learners and a specific content area (Hidi and Renninger, 2006).

Learners’ motivation can be enhanced in the virtual context. Chen et al. (2021) found that, during experienced learning assisted by XR technology, the learners had higher language learning motivation than the ones who learned with non-XR technology. Moreover, the former learners were more likely to improve their language proficiency, which is significant for their future careers with a positive attitude. By engaging in an activity in an XR context, the learners feel satisfied with the activity itself, leading to their motivation to learn in a virtual context (Deci and Ryan, 2015).

Enhanced motivation promotes learners’ active learning. XR technology brings real-life objects into a virtual context that encourages active learning. Xie et al. (2019) concluded that the realistic representations of XR tools drive learners’ active discovery in vocabulary learning. Thus, the learners can manage their behavior to maintain focus and undertake a task, and they are in control of their actions, which are self-regulation and self-efficacy in CAMIL (Makransky and Petersen, 2021).

Moreover, learners’ cognitive load can also be expanded in the virtual context. Jain et al. (2020) studied the cognitive learning environment of learning the Hindi language. They found that the virtual context could help learners comprehend the language and decrease their cognitive load when understanding the language, especially in language reading. Moreover, Dolgunsöz et al. (2018) also found that the rich information offered by the virtual world benefits the long-term cognitive load since the learners could remember details in the long run after they have acquired information with XR. Thus, the learners’ working memory capacity can be expanded with the assistance of XR technology.

The current review found that XR-based language learning leads to different learning outcomes. Of the 10 reviewed studies, 8 indicated that technological and cognitive factors lead to factual, conceptual, and procedural knowledge. Xie et al. (2019) found that learners’ pronunciation and fluency stayed stable since the learners spent more time studying and practicing the factual knowledge of language pronunciation with XR tools. Dolgunsöz et al. (2018) found that the long-term retention of XR-assisted writing performance is a good case of conceptual knowledge achievement, which indicates that the detailed information the learners acquired in the virtual world is conceptualized in their cognitive system. Wan Daud et al. (2021) designed and developed vocabulary learning materials with XR technology and illustrated how to do things with XR technology in language learning.

## 6. Conclusion

The current study summarized previous studies on XR use in language learning from a CAMIL perspective and found that the existing studies tend to focus on isolated aspects of XR-related language learning, for example, the study of technological factors, XR affordance, or the cognitive factors in XR-based language learning. Moreover, the continuum studies, from technical issues to the learning outcomes within the CAMIL, are lacking. For future studies, researchers should adopt a specific language-learning-theory perspective to examine the relationship between technological factors, XR affordance, and cognitive factors and to determine, against the relationships, how the learning outcomes can be achieved. Moreover, the factors that influence these relationships need to be explored. In particular, examining the factors that influence the relationship between technical issues and cognitive factors is necessary because of the virtual learning context. Subsequently, based on these findings, future studies should conduct more empirical studies with XR-assisted language learning and adopt language-learning theories to systematically illustrate the XR-based language-learning process.

## Author contributions

Both authors listed have made a substantial, direct, and intellectual contribution to the work, and approved it for publication.
